# Does nirsevimab prevent lower respiratory infections caused by respiratory syncytial virus?

**DOI:** 10.1038/s41372-024-01970-y

**Published:** 2024-04-18

**Authors:** Kelsey Sullivan, Brynne Sullivan

**Affiliations:** Division of Neonatology, Department of Pediatrics, University of Virginia, Charlottesville, VA USA

**Keywords:** Infectious diseases, Immunotherapy

## Manuscript citation

Hammitt LL, Dagan R, Yuan Y, et al. Nirsevimab for prevention of RSV in healthy late-preterm and term infants. N Engl J Med. 2022;386 Suppl 9:837–846. 10.1056/NEJMoa2110275.

## Question

In healthy late preterm and term infants, does a single injection of the novel monoclonal antibody nirsevimab, compared to placebo, decrease the incidence of medically-attended lower respiratory tract infections (LRTIs) caused by respiratory syncytial virus (RSV) in the first 150 days after injection?

## Methods

### Design

This is a multicenter phase 3 randomized controlled trial.

### Allocation

Participants were randomly assigned to receive nirsevimab or placebo in a 2:1 allocation. Randomization was stratified by age (<3, 3–6, or >6 months) and by hemisphere (northern vs southern).

### Blinding

Investigators, parents, and guardians were blinded to the study group assignments.

### Follow-up period

The incidence of medically-attended RSV-associated LRTI was followed through day 511 following the administration of the study drug or placebo.

### Setting

The study enrolled participants in the northern hemisphere in 2019 (150 sites in 20 countries) and the southern hemisphere in 2020 (10 sites in 1 country). Patient enrollment, particularly in the southern hemisphere, was adversely and unexpectedly affected by the COVID-19 pandemic.

### Participants

Each site obtained approval from an Institutional Review Board. For each enrolled participant, there was a signed informed consent form.

### Inclusion criteria

Infants born at 35 weeks gestation or older, who were less than one year of age at the time of enrollment, and who were entering their first RSV season were eligible.

### Exclusion criteria

Infants were excluded if they were eligible for palivizumab based on local or national criteria, if they had an acute illness within 7 days before randomization, or if they had a documented RSV infection at or before randomization.

### Intervention

Nirsevimab is a novel recombinant IgG1 monoclonal antibody that binds the F1 and F2 subunits of the RSV fusion protein. It locks the protein in the prefusion conformation and thus blocks viral entry into the host cell. Dosing of nirsevimab was 100 mg (≥5 kg) or 50 mg (<5 kg). The control group received a placebo injection.

## Outcomes

### Primary outcome

The primary efficacy outcome was medically attended RSV-associated LRTI through the first 150 days following nirsevimab injection. The case definition for medically-attended RSV-associated LRTI required a positive polymerase chain reaction test for RSV, clinical signs of LRTI (rhonchi, rales, crackles, and wheeze), and severe respiratory disease (defined as the presence of hypoxemia, new-onset apnea, retractions, grunting, nasal flaring, respiratory failure, or dehydration requiring IV fluid administration).

### Secondary outcomes

A secondary efficacy outcome was hospitalization for RSV-associated LRTI through the first 150 days following nirsevimab injection. The investigators also monitored drug pharmacokinetics, development of antidrug antibodies, and adverse events through day 511 following injection.

### Analysis and sample size

A sample size of 1500 was chosen to provide 99% power to detect a 70% lower relative risk of the primary outcome with a two-sided significance level of 0.05, assuming an incidence of 8% among control infants. Enrollment was planned to continue until the sample size required for assessment of safety was reached, which was 3000.

ClinChoice, a contract research organization, performed the data analysis. Subgroup analyses were predetermined and stratified by hemisphere, sex, weight, race, gestational age, and age at randomization. However, due to a lack of events in the southern hemisphere, the hemisphere was later removed as a covariate. Efficacy outcomes were calculated as intention-to-treat, while safety outcomes were calculated as-treated. The relative risk of the primary outcome was estimated using a Poisson regression model with robust variance, and efficacy was expressed as one minus the relative risk and given as a percentage. To minimize type 1 error, the secondary endpoint was only calculated if statistical significance was found in the primary endpoint. Multiple imputation was employed for missing data.

## Results

One thousand four hundred ninety participants were randomized and 1478 (99%) received an injection (987 nirsevimab and 491 placebo). Of these, 1465 participants were followed through 150 days. The northern hemisphere sites enrolled 1028 participants, and the South African sites enrolled 462.

## Primary outcome

The efficacy of nirsevimab in preventing medically-attended LRTI associated with RSV in the first 150 days following injection was 74.5%, with a 95% confidence interval (CI) [49.6–87.1%, *p* < 0.001]. The rate of medically-attended RSV-associated LRTI was 1.2% in the intervention group vs 5.0% in the placebo group. The number needed to treat was 1000 infants treated to prevent 55.8 cases of medically-attended LRTI associated with RSV.

## Secondary outcomes

The efficacy of nirsevimab in preventing hospitalization due to RSV-associated LRTI was 62.1% (95% CI −8.6–86.8, *p* = 0.07). A pre-specified pooled analysis combined the intention-to-treat data from this trial with data from a previous trial studying the efficacy of 50 mg of nirsevimab in infants born at 29 + 0 to 35 + 6 weeks gestation [1] and showed an efficacy of 77.3% (95% CI 50.3–89.7%, *p* < 0.001). Subgroup analysis showed lower efficacy in younger (≤3 months) and smaller (weight < 5 kg) infants.

Pharmacokinetics data demonstrated a linear decrease in serum nirsevimab concentrations over time. Antidrug antibody was detected in 58 of the 951 infants (6.1%) who received nirsevimab and 5 of the 473 (1.1%) who received placebo. The nirsevimab pharmacokinetics of patients who developed antidrug antibodies did not differ from those who did not through day 151 after injection.

Frequencies of reported adverse events were similar between the intervention and placebo groups, and most were of low severity. Serious adverse events occurred in 6.8% in the nirsevimab group and 7.3% in the placebo group. Three infants in the nirsevimab group died between days 140 and 361 after injection; no infants in the placebo group died. The investigators reviewed the deaths and did not believe that they were related to the study intervention. Overall, 1.0% of infants in the nirsevimab group and 1.4% in the placebo group experienced adverse events that were considered related to the trial regimen. None of these were considered serious adverse events.

## Study conclusion

The authors concluded that nirsevimab has good efficacy in preventing medically-attended LRTIs due to RSV in healthy late preterm and term infants. A post hoc analysis of the data from the northern hemisphere suggested that the number needed to treat to prevent one medically-attended LRTI was 11 (95% CI 9–16), and the number needed to treat to prevent one hospitalization was 57 (95% CI 31–500). Additionally, the authors concluded that nirsevimab has a good safety profile.

## Commentary

RSV is one of the most ubiquitous respiratory viruses worldwide and contributes to significant disease burden, morbidity, and mortality in infants and children [2–4]. Young infants and children, particularly those with medical complications such as prematurity or complex congenital heart disease, are at the highest risk for morbidity and mortality from LRTI associated with RSV [5, 6]. Despite the increased risk for children with medical complexity, most medically-attended RSV LRTIs and hospitalizations occur in otherwise healthy children [6].

The widespread use of nirsevimab promises to reduce the burden of RSV significantly among young children [7]. For this reason, the FDA recently approved nirsevimab for use in neonates and infants entering their first respiratory season and for children up to 24 months with increased risk for severe RSV-related respiratory disease entering their second respiratory season [8]. The American Academy of Pediatrics and the Centers for Disease Control Advisory Committee on Immunization Practices recommended the use of nirsevimab in all infants <8 months and in infants and children 8–19 months who are at high risk of severe RSV related disease for the 2023–2024 RSV season [9, 10].

Several studies have examined the efficacy and safety of nirsevimab [1, 11, 12]. The manuscript discussed in this review reports the results of the MELODY Phase III clinical trial (ClinicalTrials.gov number NCT03979313) [11]. At the time of publication, the investigators had acquired sufficient data to determine the primary endpoint. However, they had only half the planned enrollment for examining secondary outcomes. They continued enrollment and published the updated results [12]. Additionally, the authors report results from the analysis of pooled data from the previous Phase IIb placebo-controlled trial, which examined the safety and efficacy of nirsevimab in healthy preterm infants (ClinicalTrials.gov number NCT02878330) [1]. This practice of pooling data from the Phase IIb and Phase III trials is not typically performed but is advantageous in the process of developing treatments for rare diseases [13]. Although the authors did not explicitly justify using this technique in this common disease, analysis of the pooled data is compelling given the unanticipated effect of the COVID-19 pandemic on enrollment.

During the first year of the COVID-19 pandemic, countries observed reduced RSV transmission followed by increased out-of-season transmission. After enrollment began in the southern hemisphere, there were no cases of medically-attended RSV-associated LRTI during the first 151 days after injection, but there were increased out-of-season cases between days 152 and 361 following injection. An additional potential weakness of this study is that the assessment of adverse events following study injection was conducted by the investigators rather than by an independent reviewer. Despite these limitations, the results of the MELODY trial support the use of nirsevimab to reduce the burden of RSV-associated LRTI in infants.

## EBM lesson: unbalanced allocation (e.g., 2:1) in a randomized trial

This study utilized unbalanced allocation in a 2:1 ratio when randomizing study participants to intervention vs placebo. Unbalanced allocation is frequently employed in confirmatory trials, but remains somewhat controversial [14].

Unbalanced allocation has several potential benefits. From a statistical standpoint, 1:1 allocation is the most efficient approach because it requires the fewest study participants to achieve adequate power [15]; a study employing a 2:1 allocation ratio requires 12% more participants in order to achieve equal power to a 1:1 allocation, and this number increases to 33% more participants in a 3:1 allocation scheme [14]. However, one potential statistical benefit is that, at modest ratios (up to 3:1), unbalanced allocation can retain statistical power to determine the efficacy of a novel intervention while exposing a greater proportion of trial participants to the study intervention, which may increase the likelihood of detecting rare adverse outcomes [14, 15]. A second potential benefit, some argue, is that unbalanced allocation may enhance study enrollment when the participants desire one study arm more than the other, though there is no evidence to support this argument [16]. Additionally, some contend that investigators may favor unequal allocation when a pilot study has demonstrated promising efficacy of an intervention and yet further study is required for confirmation; the rationale behind this thought is that in such cases, clinicians conducting a confirmatory trial may have reduced equipoise as compared to the general population and thus desire that a higher percentage of participants receive the intervention [15].

In contrast, there are also several potential drawbacks to unbalanced allocation. Unbalanced allocation limits the statistical power of a study at any ratio above 1:1 [14, 15]; higher ratios require considerably higher enrollment, which reduces efficiency, exposes a higher number of participants to treatments of unknown efficacy and/or safety, and increases cost [14]. An ethical concern is that unequal allocation may falsely convey an impression of a therapeutic advantage in the intervention arm, which may unduly influence subjects to enroll based on an expectation of being randomized to the intervention [14]. Overall, the unequal allocation has potential benefits and drawbacks. It is important for investigators to understand the impact of unbalanced allocation when considering this technique and to justify its use.

## Data Availability

Data sharing is not applicable to this article as no data sets were generated or analyzed. The MEDLEY and MELODY trials have had multiple publications associated with them.
